# Comprehensive N-Glycan Profiling of Avian Immunoglobulin Y

**DOI:** 10.1371/journal.pone.0159859

**Published:** 2016-07-26

**Authors:** Sarah Gilgunn, Silvia Millán Martín, Mark R. Wormald, Julia Zapatero-Rodríguez, Paul J. Conroy, Richard J. O’Kennedy, Pauline M. Rudd, Radka Saldova

**Affiliations:** 1 School of Biotechnology, Dublin City University, Dublin 9, Ireland; 2 Biomedical Diagnostics Institute, National Centre for Sensor Research, Dublin City University, Dublin 9, Ireland; 3 NIBRT GlycoScience Group, National Institute for Bioprocessing Research and Training, Fosters Avenue, Mount Merrion, Blackrock, Dublin 4, Ireland; 4 Oxford Glycobiology Institute, Department of Biochemistry, University of Oxford, South Parks Road, Oxford OX1 3QU, United Kingdom; 5 Department of Biochemistry and Molecular Biology, Faculty of Medicine, Nursing and Health Science, Monash University, Melbourne, VIC 3800, Australia; 6 ARC Centre of Excellence in Advanced Molecular Imaging, Monash University, Melbourne, VIC 3800, Australia; University of Pavia, ITALY

## Abstract

Recent exploitation of the avian immune system has highlighted its suitability for the generation of high-quality, high-affinity antibodies to a wide range of antigens for a number of therapeutic and biotechnological applications. The glycosylation profile of potential immunoglobulin therapeutics is species specific and is heavily influenced by the cell-line/culture conditions used for production. Hence, knowledge of the carbohydrate moieties present on immunoglobulins is essential as certain glycan structures can adversely impact their physicochemical and biological properties. This study describes the detailed *N-*glycan profile of IgY polyclonal antibodies from the serum of leghorn chickens using a fully quantitative high-throughput *N*-glycan analysis approach, based on ultra-performance liquid chromatography (UPLC) separation of released glycans. Structural assignments revealed serum IgY to contain complex bi-, tri- and tetra-antennary glycans with or without core fucose and bisects, hybrid and high mannose glycans. High sialic acid content was also observed, with the presence of rare sialic acid structures, likely polysialic acids. It is concluded that IgY is heavily decorated with complex glycans; however, no known non-human or immunogenic glycans were identified. Thus, IgY is a potentially promising candidate for immunoglobulin-based therapies for the treatment of various infectious diseases.

## Introduction

Antibodies are at the forefront of the field of targeted therapeutics and diagnostics due to their natural high affinity and excellent half-life properties [1]. These molecules can be readily manipulated using standard molecular biology techniques into specialised antibodies that are tailored to perform efficiently in their chosen end-point application [2]. The biopharmaceutical industry has heavily invested in antibody-based therapeutics, which currently represents the largest and fastest growing class of biopharmaceuticals [3].

Polyclonal and recombinant antibodies are developed in many different species. However, a large number of protein targets are highly conserved in mammalian evolution and commonly used mammalian species, such as rabbits and mice, are thus inclined to render a somewhat limited immune response due to immunological tolerance invoked during foetal development [4]. The use of a species more phylogenetically distant from humans such as chickens, who diverged from mammalian genomes some 310 million years ago [5], are ideal alternatives for immunisation and selection of antibodies against highly conserved human proteins [4,6].

**Fig 1:**
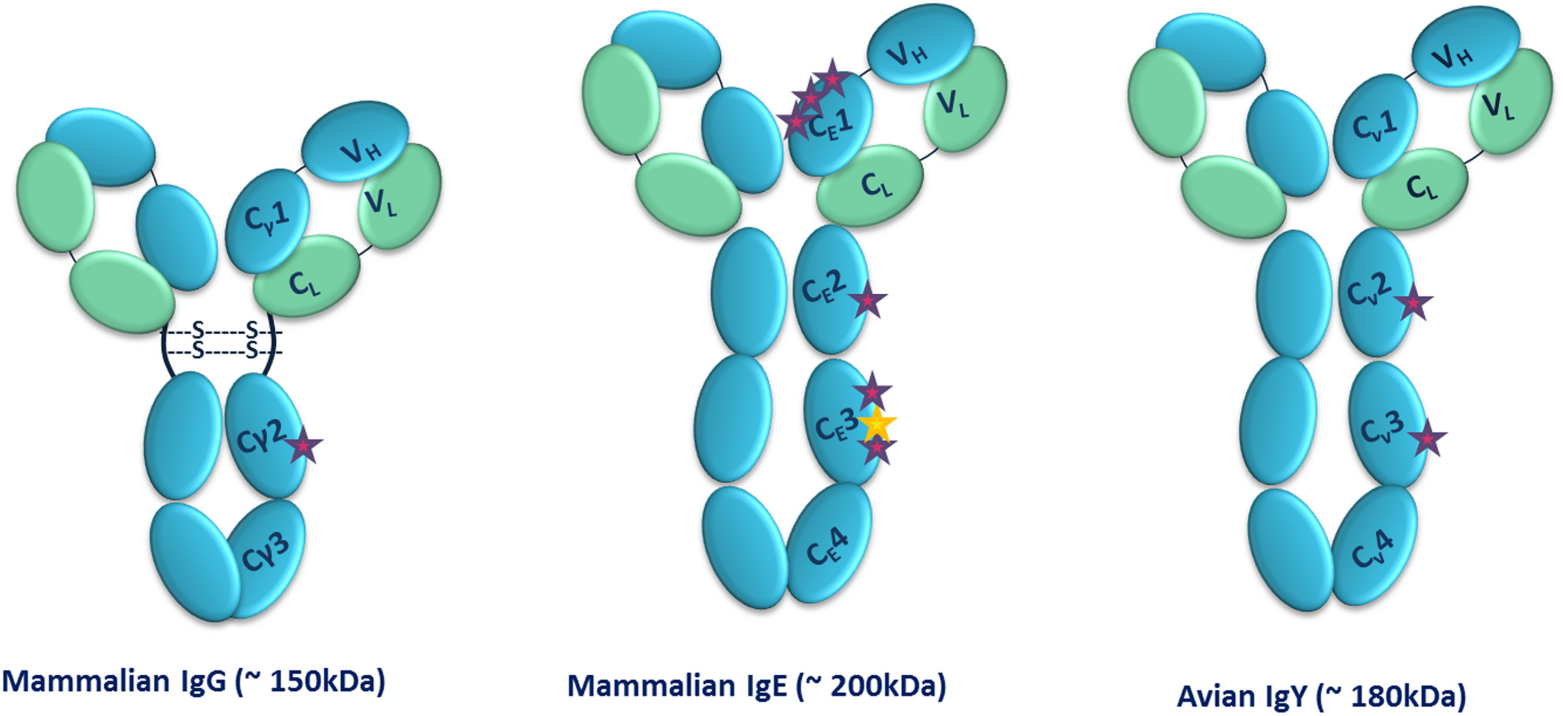
Structures of Immunoglobulin’s G, E and Y and their *N*-glycosylation sites. IgG (left) is composed of 2 identical heavy chains that each comprise a variable domain (V_H_) and three constant domains (C_γ_1, C_γ_2 and C_γ_3) with a single carbohydrate site (purple star). In contrast, the additional constant domain in IgE (middle) is much more heavily glycosylated than IgG, with 7 *N-*glycosylation sites, however, one site (Asn 264) is unoccupied (yellow star) [7]. IgY is also comprised of four constant domains per heavy chain (Cv1-Cv4), with two carbohydrate sites (right). The flexible hinge region found in IgG is absent in IgE and IgY and thus may restrict their flexibility in comparison to IgG.

IgY is the predominant serum immunoglobulin in birds, reptiles and amphibians and is considered to be evolutionary ancestor of uniquely mammalian IgG and IgE antibodies [6]. Although IgY has characteristics and functions similar to its mammalian counterpart, IgG, with 2 heavy (67–70 kDa each) and two light (25 kDa each) chains (Fig 1), structural differences exist in the number of constant heavy domains, as IgY has an additional constant heavy domain resulting in its higher molecular mass (180 kDa). Furthermore, IgY lacks of a hinge region and has significantly reduced flexibility in comparison to IgG. This limited flexibility is derived from proline-glycine rich regions around the Cν1-Cν2 and Cν2-Cν3 domains [6]. These structural differences provide IgY with distinct biochemical properties and behaviour (Table 1).

**Table 1:** Comparison of the different properties of IgG and IgY.

	IgG	IgE	IgY
**Species**	Mammals	Mammals	Birds, reptiles, amphibians and lungfish
**Molecular weight (kDa)**	150	200	180
**Isoelectric point (pI)**	6.4–9.0	5.2–5.8	5.7–7.6
**Concentration (mg/mL)**	10–12 (serum)	10^−4^	8–10 (Serum) 15–25 (Egg Yolk)
**Number of constant domains**	4 (3 H and 1 L)	5 (4 H and 1 L)	5 (4 H and 1 L)
**Hinge**	Yes	No	No
**Complement binding**	Yes	No	No
**Rheumatoid factor binding**	Yes	No	No
**Fc receptor binding**	Yes	No	No
**Mediates anaphylaxis**	No	Yes	Yes
**Binding to protein A/G**	Yes	No	No

IgY is more heavily glycosylated than its mammalian counterpart as it contains two potential *N-*glycosylation sites. One is located in Cv3 domain, that is absent in the mammalian IgG, and the other is located in the Cv3 domain which corresponds to the C_H_2 (Cγ2) domain of mammalian IgG (Fig 1) [6,8]. Structural characterisation of *N-*glycans present on antibody therapeutics is a regulatory requirement as the nature of these glycans can decisively influence the therapeutic performance of an antibody [9]. The linked carbohydrate moieties of therapeutic antibodies affect both their thermal stability and physicochemical properties, along with other crucial features like receptor-binding activity, circulating half-life and immunogenicity [10]. *N-*glycan profiling of therapeutic antibodies with good reproducibility is vital to fulfil the needs of the both the biopharmaceutical industry and national regulatory agency requirements [11].

There is now considerable awareness of the therapeutic value of IgY antibodies with respect to a variety of pathologies including, but not limited to, pulmonary or gastrointestinal infections. For a detailed review of the current IgY therapeutic approaches in both animal studies and clinical trials in human cohorts see Spillner *et al*., 2012 [1].

The *N*-glycosylation pattern of avian IgY was previously shown to be more analogous to that in mammalian IgE than IgG, presumably reflecting the structural similarity to mammalian IgE [7]. While previous studies have elucidated the IgY *N*-glycan profile in detail from serum, egg yolk and various other expression vehicles [8,12,13], in this study, the chromatography technique was significantly improved through the use of hydrophilic interaction chromatography ultra-performance liquid chromatography (HILIC UPLC) which allows for shorter run times and greatly increased resolution. In addition, the bioinformatics tool, GlycoBase, was used to greatly assist the analyses. GlycoBase consists of a database with HILIC and mass spectrometry data for over 460 2-AB-labeled *N*-linked, 68 O-linked, and 71 free glycan structures. This reliable and robust method facilitates detailed analysis of femtomolar quantities of N-linked sugars released from glycoproteins

This study describes the detailed *N-*glycan profile of IgY polyclonal antibodies from the serum of Leghorn chickens using a fully quantitative high-throughput *N-*glycan analysis based on ultra-performance liquid chromatography (UPLC) separation of released glycans.

## Materials and Methods

### Ethics Statement

This study was approved by the Research Ethics Committee of Dublin City University (Approval Ref. DCUREC/2011/016) and all procedures in which animals were used were carried out in accordance with licence B100/2705 granted by the Minister for Health of Ireland under the Cruelty to Animals Act 1876 (as amended). The minimum number of animals was used, and that chicken was socially housed under environmentally controlled conditions for the duration of the study. The procedures were classified as "mild", and all procedures were carried out by highly trained, competent, personnel.

### Immunoglobulin Y purification

Polyclonal IgY antibodies were purified from the serum of an adult female leghorn chicken using the Pierce^®^ Thiophilic Adsorption Kit (Thermo Scientific, Ireland). This protocol was carried out as per manufacturer’s guidelines and the eluted protein fractions were pooled and concentrated in a 10kDa MWCO Vivaspin column (Sartorius, Ireland). Total protein concentration was measured by spectrophotometry at 280 nm (NanoDrop 1000). The purified IgY polyclonal antibody sample was subjected to sodium dodecyl sulfate-polyacrylamide gel electrophoresis (SDS-PAGE) in a 12% gel and stained with InstantBlue (MyBio, Ireland). The sample was also subjected to SDS-PAGE in a 12% gel and subsequently blotted onto a nitrocellulose membrane using the Pierce G2 Fast Blotter system (Thermo Scientific, Ireland). The membrane was blocked for 1 hour at room temperature with PBS containing 5% (w/v) skim milk. To detect the heavy and light chains of the IgY, the sample was probed with a horse radish peroxidase (HRP) labelled-donkey anti- IgY (H+L)-specific antibody (Gallus Immunotech, Canada) (1:2,000 dilution) in PBS-Tween 20 (0.05%, v/v)–skim milk (1%, w/v) for 2 hours at room temperature. Specific bands were visualised using liquid TMB as a substrate for HRP.

### Glycan release

*N-*glycans were released from the dissolved samples (1 mg/mL) using PNGase F, (100 mU/mL; Prozyme, San Leandro, CA, USA) in accordance with methods previously described by Royle *et al*., 2006 and Royle *et al*,. 2008 [14,15]. Briefly, samples were reduced and alkylated, immobilized in SDS-gel blocks in 96 well plates and then washed. Released glycans were then fluorescently labelled with 2-aminobenzamide (2AB) by reductive amination [16]. Excess 2AB reagent was removed by ascending chromatography on Whatman 3MM paper in acetonitrile.

### Ultra-Performance Liquid Chromatography (UPLC)

2AB derivatized *N-*glycans were separated by UPLC with fluorescence detection on a Waters Acquity UPLC H-Class instrument consisting of a binary solvent manager, sample manager and fluorescence detector under the control of Empower 3 chromatography workstation software (Waters, Milford, MA, USA). The hydrophilic interaction liquid chromatography (HILIC) separations were performed using a Waters Ethylene Bridged Hybrid (BEH) Glycan column, 150 x 2.1 mm i.d., 1.7 μm BEH particles, with 50 mM ammonium formate, pH 4.4, as solvent A and acetonitrile (MeCN) as solvent B. The 30 minute method was used with a linear gradient of 30–47% (v/v) with buffer A at 0.56 mL/minute flow rate for 23 minutes followed by 47–70% (v/v) A and finally reverting back to 30% (v/v) A to complete the run [17]. An injection volume of 10 μL sample prepared in 70% (v/v) MeCN was used throughout. Samples were maintained at 5°C prior to injection, while separation was carried out at 40°C. The fluorescence detection excitation/emission wavelengths were λ_excitation_ = 330 and λ_emission_ = 420 nm, respectively. The system was calibrated using an external standard of hydrolysed and 2AB-labeled glucose oligomers to create a dextran ladder, as described previously [14].

### Weak Anion-Exchange High-Performance Liquid Chromatography (WAX HPLC)

Weak anion exchange (WAX) HPLC to separate the *N*-glycans by charge was carried out as detailed in Royle *et al*., 2006, with a fetuin *N-*glycan standard as reference. WAX HPLC was performed using a Vydac 301VHP575 7.5 × 50 mm column (Anachem) on a 2695 Alliance separations module with a 2475 fluorescence detector (Waters), which was set with detection excitation/emission wavelengths of λ_excitation_ = 330 and λ_emission_ = 420 nm, respectively [15]. Solvent A was 0.5 M formic acid adjusted to pH 9.0 with ammonia solution, and solvent B was 10% (v/v) methanol in water. Gradient conditions were as follows: a linear gradient of 0 to 5% (v/v) A over 12 minutes at a flow rate of 1 mL/minute, followed by 5–21% (v/v) A over 13 minutes and then 2–50% (v/v) A over 25 minutes, 80–100% (v/v) A over 5 minutes followed by 5 minutes at 100% A. Samples were prepared in water and a fetuin *N*-glycan standard was used for calibration [15].

### Exoglycosidase digestions

Exoglycosidase digestions were carried out on aliquots of the total labelled *N-*glycan pools in accordance with methods previously described by Royle *et al*., 2006 [14] and according to manufacturer’s instructions. All exoglycosidase enzymes were obtained from Prozyme, San Leandro, CA, USA. The 2AB-labeled glycans were digested in a volume of 10 μL for 18 hours at 37°C in 50 mM sodium acetate buffer, pH 5.5 (except in the case of jack bean α-mannosidase (JBM) where the buffer was 100 mM sodium acetate, 2 mM Zn^2+^, pH 5.0), using arrays of the following enzymes: *Arthrobacter ureafaciens* sialidase (ABS), 0.5 U/mL; *Streptococcus pneumoniae* sialidase (NAN1), 1 U/mL; bovine testes β-galactosidase (BTG), 1 U/mL; *Streptococcus pneumoniae* β-galactosidase (SPG), 0.4 U/mL; bovine kidney α-fucosidase (BKF), 1 U/mL; β-*N*-Acetylglucosaminidase cloned from *S*. *pneumonia*, expressed in *Escherichia coli* (GUH), 8 U/mL and JBM, 60 U/mL. After incubation, enzymes were removed by filtration through a 10 kDa protein-binding EZ filters (Millipore Corporation) [15]. *N-*Glycans were then analysed by HILIC UPLC and WAX HPLC.

### Ultra-Performance Liquid Chromatography -Fluorescence-Mass Spectrometry (UPLC-FLR-MS)

For UPLC-FLR-QTOF MS analysis lyophilised IgY samples were reconstituted in 3 μl of water and 9 μl acetonitrile. Online coupled fluorescence (FLR)-mass spectrometry detection was performed using a Waters Xevo G2 QTof—with Acquity^®^ UPLC (Waters Corporation, Milford, MA, USA) and BEH Glycan column (1.0 x 150mm, 1.7 μm particle size).

For MS acquisition data the instrument was operated in negative-sensitivity mode with a capillary voltage of 1.80 kV. The ion source block and nitrogen desolvation gas temperatures were set at 120°C and 400°C, respectively. The desolvation gas was set to a flow rate of 600 L/h. The cone voltage was maintained at 50V. Full-scan data for glycans were acquired over *m/z* range of 450 to 2500. Data collection and processing were controlled by MassLynx 4.1 software (Waters Corporation, Milford, MA, USA).

The fluorescence detector settings were as follows: λ_excitation_: 330 nm, λ_emission_: 420 nm; data rate was 1pts/second and a PMT gain = 10. Sample injection volume was 10 μL. The flow rate was 0.150 mL/minute and column temperature was maintained at 60°C; solvent A was 50 mM ammonium formate in water (pH 4.4) and solvent B was acetonitrile. A 40 minute linear gradient was used and was as follows: 28% (v/v) A for 1 minute, 28–43% (v/v) A for 30 minutes, 43–70% (v/v) A for 1 minute, 70% (v/v) A for 3 minutes, 70–28% (v/v) solvent A for 1 minute and finally 28% (v/v) A for 4 minutes.

Samples were diluted in 75% (v/v) acetonitrile prior to analysis. The weak wash solvent was 80% (v/v) acetonitrile and the strong wash solvent was 20% (v/v) acetonitrile. To avoid contamination of Mass Spec system, flow was sent to waste for the first 1.2 minutes and after 32 minutes.

### Molecular Modelling of IgY

Molecular modelling of IgY was performed on a Silicon Graphics Fuel workstation using InsightII and Discover software (Accelrys Inc., San Diego, USA). Figures were produced using the program Pymol [18]. Protein structures used for modelling were obtained from the Protein Data Bank (PDB) database [19]. The peptide structure of chicken IgY was based on the crystal structures of human IgE domains Cε2–4[20] and human IgG Fab domain [21]. Sequence alignment and methods for generation of homology model are provided in S1 File.

## Results

### IgY Purification

**Fig 2:**
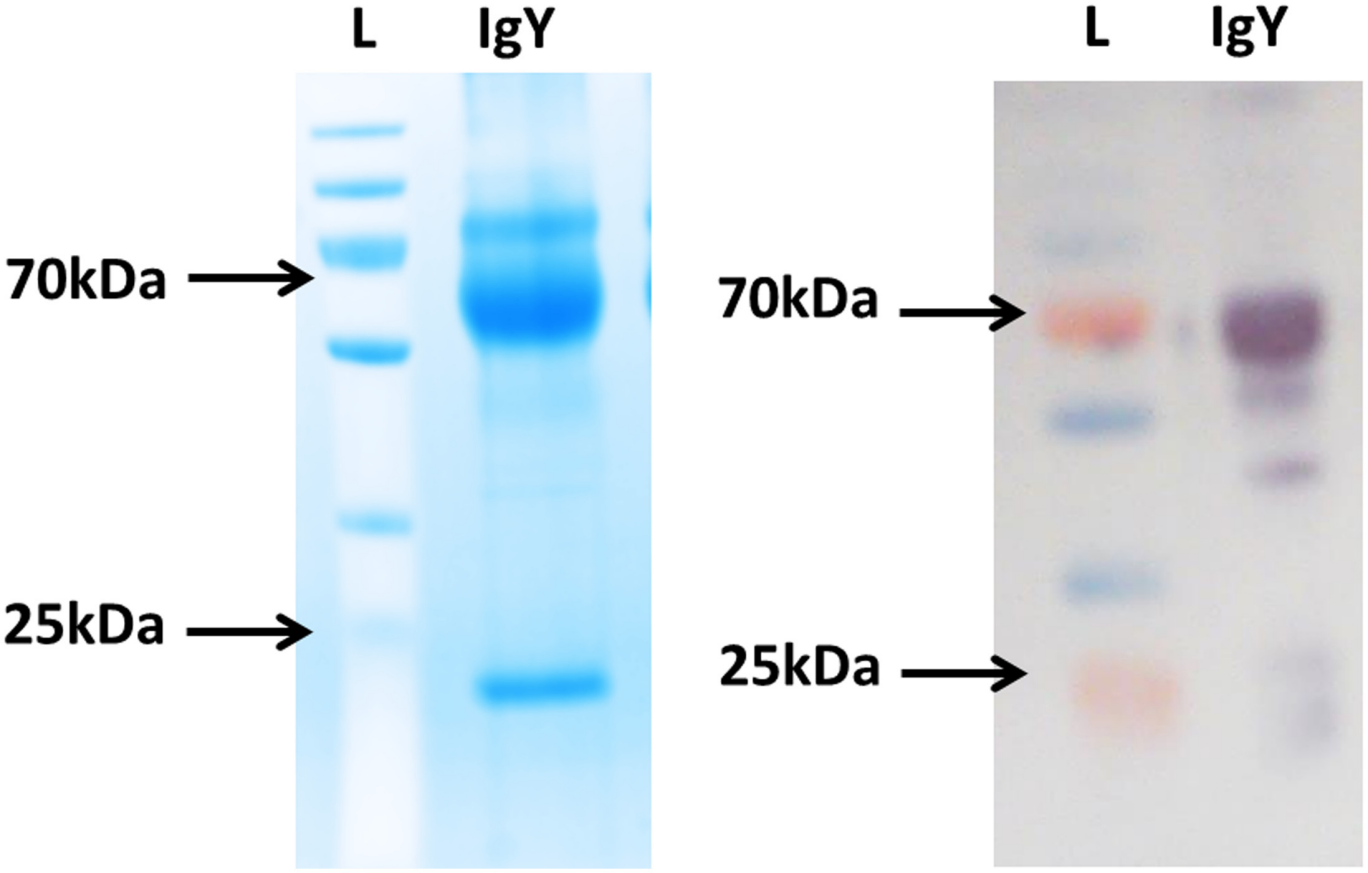
IgY Purification. IgY purified from chicken serum was resolved on 12% SDS-PAGE gels and visualised by staining with InstantBlue (left). The resolved proteins were also transferred to nitrocellulose membranes and the presence of the heavy chains at approximately 65–68 kDa and the light chains at 25 kDa can be seen after probing with an anti-IgY H+L-HRP-tagged antibody (right). L: PageRuler Plus Prestained protein ladder.

Immunoglobulin Y differs from most of the other immunoglobulins as it does not bind protein A or protein G [22]. Here, IgY was successfully recovered from the serum of chickens using thiophilic adsorption, which is based on the principles of hydrophobic interaction chromatography. Many proteins, particularly immunoglobulins will bind to an immobilised ligand that contains a sulfone group neighbouring a thioether. Addition of salts such as potassium sulphate will promote binding by encouraging the protein into close proximity of the ligand [23,24]. Total protein concentration was determined to be 11.5 mg/mL by spectrophotometry at 280 nm (NanoDrop 1000). The heavy and light chains of the purified IgY were visualized by Western Blot analysis (Fig 2).

### IgY *N*-glycan profiling

The *N-*glycans released from the purified IgY were analysed by WAX HPLC and HILIC UPLC in combination with exoglycosidase digestions and structural assignments using established methods [15] and the software tool GlycoBase (https://glycobase.nibrt.ie). UPLC-FLR-QTOF MS analysis was also carried out for comparative analysis. Annotation of the *N*-glycans present in each chromatographic peak was based upon the oligosaccharide composition as derived from the *m/z* value.

**Fig 3:**
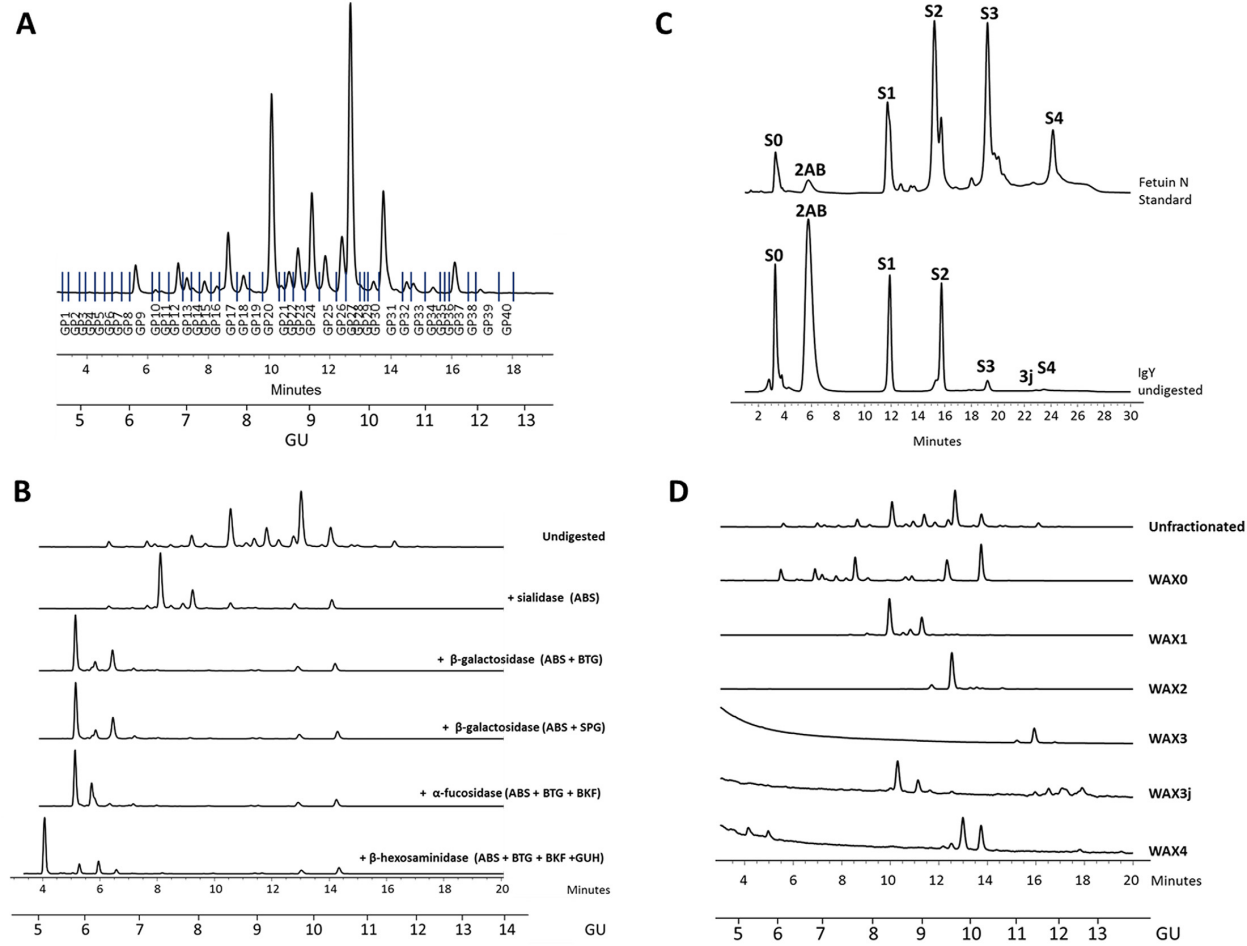
IgY *N*-glycan assignment. (A) HILIC UPLC profile of undigested *N*-glycans from serum IgY. Profiles are standardised against a dextran hydrolysate (GU). The HILIC chromatogram was separated into 40 peaks. (B) Unfractionated IgY profile was subjected to exoglycosidase digestions. (C) IgY glycans were separated according number of sialic acids on WAX HPLC and (D) each WAX fraction was then subjected to HILIC UPLC (Final IgY Structural assignments are listed S1 Table).

**Fig 4:**
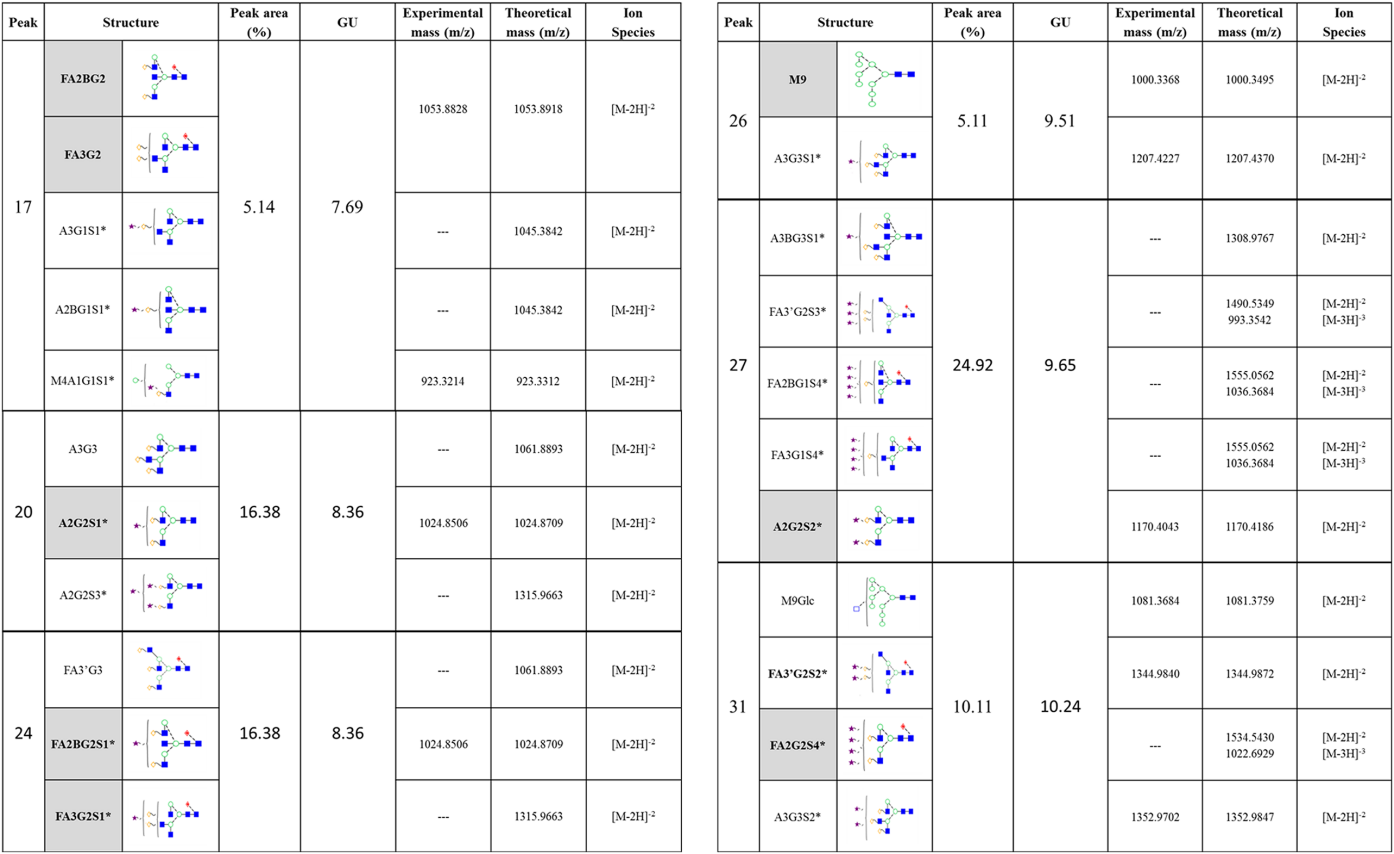
Summary of *N-*glycans identified from IgY purified form avian serum. Summary of *N*-glycans released from IgY purified from serum. The HILIC chromatogram was separated into 40 peaks and structural assignment carried made using established methods (Royle *et al*., 2008) and the software tool GlycoBase (https://glycobase.nibrt.ie). Nomenclature used is according to Royle *et al*., 2008 and Harvey *et al*., 2009 [15,25]. Shown here are the most abundant glycans identified–glycans assigned to peaks with % area great than 5%- highlighted in grey is the most abundant glycan(s) within that particular peak. For full glycan assignment see S1 Table. *Glycan nomenclature.

Over 80 different glycans structures were assigned to 40 peaks (each peak contains one or more glycans) (Figs 3 and 4 and S1 Table). *N-*glycan structures annotated include high mannose, hybrid and complex glycans with variable degrees of core fucosylation, galactosylation and sialylation. To assign the complex sialylation properly, the *N*-glycome was separated by WAX HPLC according to the number of sialic acids and then each WAX fraction was subjected to an array of sialidases for assignment of sialic acids linkages (S1 Table). The resulting HILIC UPLC profiles were combined with other exoglycosidase digests and indicated the presence of complex glycans, with more sialic acids than branches (Fig 4, S1 Table).

*Sialic acid linkages; WAX fractions were separated out into 5 fractions; S1: Monosialylated, S2: Disialylated, S3: Trisialylated, S3J: Trisialylated and S4: Tetrasialylated. All monosialylated glycans are linked by α2–3 and α2–6; Disialylated glycans have all combinations of α2–3 and α2–6 linkages [i.e. (3, 3), (3, 6) and (6, 6)]; Trisialylated glycans have all combinations of α2–3 and α2–6 linkages except (6, 6, 6) [i.e. (3, 3, 3), (3, 3, 6) and (3, 6, 6)]; and Tetrasialylated glycans have all combinations of α2–3 and α2–6 linkages [i.e. (3,3,3,3), (3,3,3,6), (3,3,6,6) and (6,6,6,6)]. Structure abbreviations: all *N*-glycans have two core GlcNAcs; F at the start of the abbreviation indicates a core-fucose α1,6-linked to the inner GlcNAc; Mx, number (x) of mannose on core GlcNAcs; Ax, number of antenna (GlcNAc) on trimannosyl core; A2, biantennary with both GlcNAcs as β1,2-linked; A3, triantennary with a GlcNAc linked β1,2 to both mannose and the third GlcNAc linked β1,4 to the α1,3 linked mannose; A3’, isomer with the third GlcNAc linked β1–6 to the α1–6 linked mannose; A4, GlcNAcs linked as A3 with additional GlcNAc β1,6 linked to α1,6 mannose; B, bisecting GlcNAc linked β1,4 to β1,3 mannose; Gx, number (x) of β1,4 linked galactose on antenna; F(x), number (x) of fucose linked α1,3 to antenna GlcNAc; Sx, number (x) of sialic acids linked mostly to galactose (Structures with more sialic acids then galactoses may have some sialic acids linked to GlcNAc or another sialic acid in form of polysialic acid.).

### Molecular Modelling of IgY

**Fig 5:**
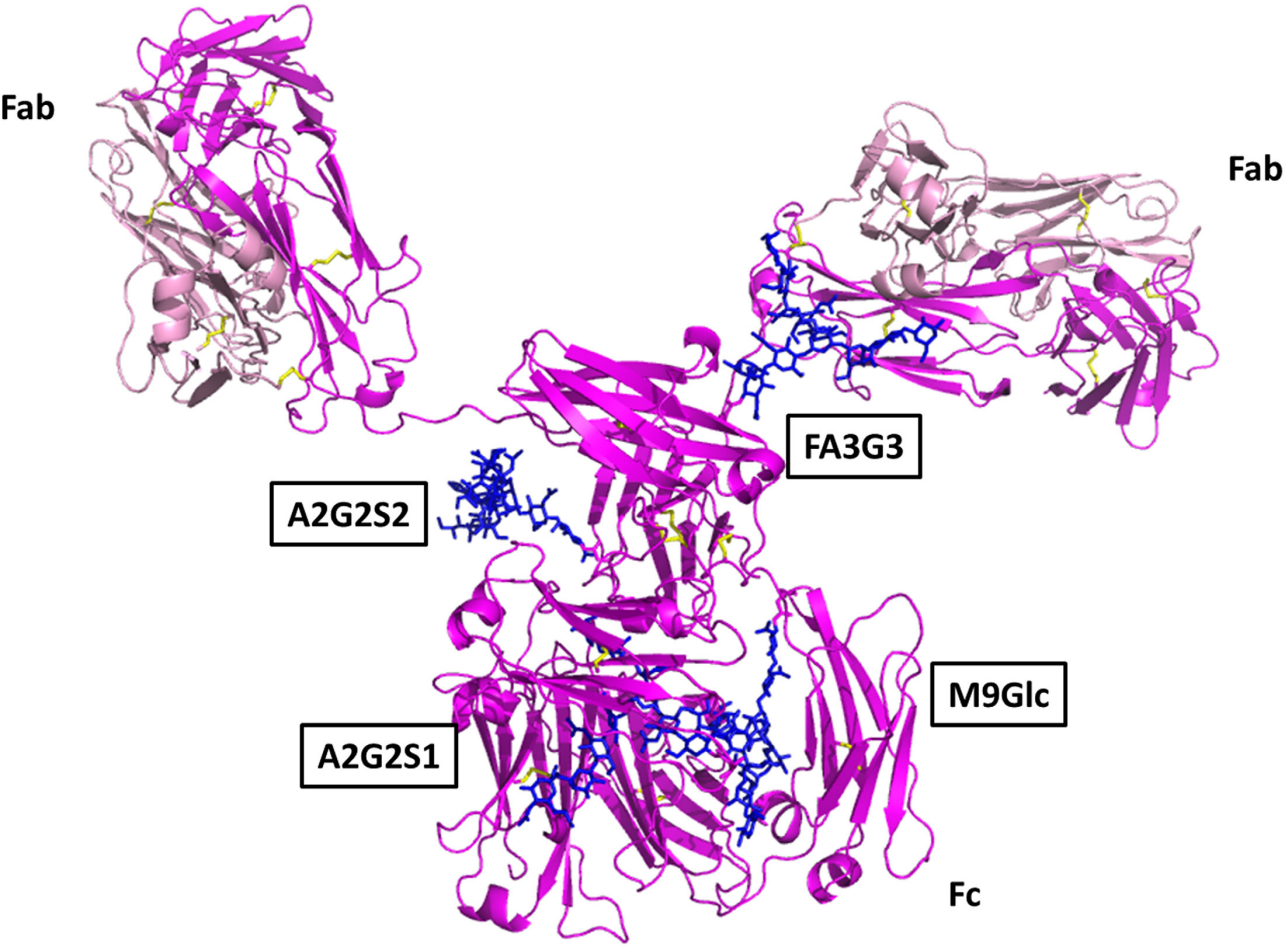
Molecular model of glycosylated IgY. Magenta—heavy chain; pink—light chain; yellow—stick representation of cysteine residues; blue—glycans. The glycans are labelled according to the nomenclature in Fig 4.

The precise glycans chosen for sites N390 (M9Glc/peak 31 and A2G2S1/peak 20) were based on the site specific glycan analysis of IgY [8]. The glycans chosen for sites N292 (A2G2S2/peak 27 and FA3G3/peak 24) were representatives from the other largest peaks. Glycan structures were generated using the database of glycosidic linkage conformations [26] and *in vacuo* energy minimisation to relieve unfavourable steric interactions. The Asn-GlcNAc linkage conformations were based on the observed range of crystallographic values [27], the torsion angles around the Asn Cα-Cβ and Cβ-Cγ bonds then being adjusted to eliminate unfavourable steric interactions between the glycans and the protein surface (Fig 5). The complete IgY sequence is provided in S1 Fig.

## Discussion

Chicken antibodies have several distinct biochemical advantages over mammalian antibodies and are widely utilised in the field of biotechnology. They do not activate the mammalian complement system nor interact with rheumatoid factors, or bacterial and human Fc receptors. Hence IgY antibodies make ideal regents for immunological assays as they can reduce assay interference in a mammalian serum sample, resulting in increased sensitivity as well as decreased background [28].

The use of chickens as hosts for the generation of therapeutic antibodies is becoming increasingly more prevalent with a greater understanding of the unique attributes of avian antibodies [2,29]. Polyclonal IgY represents an attractive approach to immunotherapy for the treatment of numerous diseases [1]. Notably, orally administered IgY preparations have been demonstrated as an alternative to antibiotics for the prevention of pulmonary *Pseudomonas aeruginosa (PA)* infections in a group of patients with cystic fibrosis [30,31]. In this study, the authors show that the IgY treated group had significantly less incidents of colonization with *PA* than the control group and none of the IgY-treated patients became chronically colonized with *PA* [30,31].

The robust, reliable methods employed in this study allow for shorter run times with increased resolution that enables identification of glycans that may not have been previously observed in other IgY glycoprofiling studies. Our structural assignments revealed serum IgY to contain mainly complex, bi-, tri- and tetraantennary glycans with or without core fucose and bisects, all with varying levels of galactosylation and sialylation, hybrid and high mannose glycans.

In investigating the site-specific *N-*glycosylation of IgY, Suzuki and Lee [8] noted the Fc portion of IgY possesses a *N-*glycosylation site which is structurally equivalent to conserved glycosylation sites of other Ig classes in mammals and is composed of predominantly high-mannose type oligosaccharides [8]. This uniquely avian glycosylation pattern at the conserved *N-*glycosylation site is thought to be attributed to the structural differences between IgG and IgY (IgY lacks the defined hinge region observed in IgG) [12]. The additional *N-*glycosylation site, located in Cv2 domain, was previously shown to contain exclusively complex-type oligosaccharides [8,12]. These distinct avian glycosylation patterns and structural differences provide IgY with unique biochemical properties and behaviour. A model of IgY with the glycans identified from this study was generated following glycan assignment (Fig 5). Characterisation of the individual glycans decorated on a protein is essential for detailed understanding of structure/function relationships and the design of potential therapeutic agents. The model generated from this study aims to enhance our understanding of the therapeutic potential of IgY. Computational modelling methods are universally accepted as central tools in the invention process for many biopharmaceuticals, facilitating drug development areas, such as optimising affinity for a target while minimising cross reactive effects, alongside optimising pharmacokinetic properties [32].

The oligosaccharide content of therapeutic immunoglobulins plays a significant role in its bioactivity and pharmacokinetic (PK) activity. Raju and colleagues (2000) examined at variations between the glycan content of IgG across several species. These authors highlighted the importance of choosing the right host in generating therapeutic IgG as the terminal sialylation of IgG is species specific [13]. In this study, LC-MS analysis of chicken IgY suggested the *N*-linked glycosylation of chicken IgY is considerably more heterogeneous than in human IgG. Our results are consistent with previous *N*-glycan studies of IgY from both serum and egg yolk [8,12,13] and also detect several previously unidentified structures.

In this study high sialic acid content was observed, with many sialic acid isomers (same composition but different sialic acid linkage arrangements resulting in a different GU from the original structure). The presence of unusual sialic acids was also noted, which is likely to be polysialic or Sialic acid linked on *N*-Acetylgalactosamine (GalNAc) as well as on Galactose. Sialic acids are most commonly α2–3 or α2–6 linked to galactose (Gal) or α 2–6 linked to GalNAc. However, Sialic acid can also be found linked to *N*-Acetylglucosamine (GlcNAc) or to another Sialic acid in α2–8 or α2–9 linkage [33]. Polysialic acids occupy internal positions within glycans, the most common being one Sialic acid residue attached to another, often at the C-8 position [34].

The high sialic acid content of IgY is very important when considering IgY as a therapeutic agent as the level of sialic acid can have a significant impact on the PK of therapeutic antibodies. A lower content of total sialic acids can significantly reduce the half-life of a drug [35]. Hence, the high sialic acid content of IgY that was observed suggests IgY-based biotherapeutics could have potentially extended circulating half-lives and are promising candidates against a variety of pathogens. High mannose glycans were also found on the IgY, which can be removed from circulation by mannose-binding receptor, therefore lowering its half-life [36]. However, the high mannose glycans on IgY are rather low in quantity in comparison to the highly sialylated complex glycans and should have no effect on the therapeutic application of IgY. Certain glycan structures have a direct impact on the immunogenicity of therapeutic proteins, that is, their presence can affect protein structure in such a way that the protein becomes immunogenic. However the glycan structure itself can also induce an immune response. The sialic acid *N-*Glycolylneuraminic acid (Neu5Gc) and terminal galactose-α-1,3-galactose are examples of such structures that are not naturally present in humans and are known to be immunogenic when used as therapeutics [37]. These non-human antigenic structures could promote clearance of a biopharmaceutical preparation from circulation [38–40]. The chimeric mouse–human IgG1 monoclonal antibody, Cetuximab, is an anti-human epidermal growth factor receptor (EGFR) antibody used for the treatment several cancers [38]. High incidences of hypersensitivity reactions to Cetuximab were reported and a study by Chung and colleagues showed that the majority of patients who had a hypersensitivity reaction to Cetuximab also had circulating IgE antibodies against Cetuximab before therapy was initiated. These antibodies were specific for the glycan structure galactose-α-1,- 3-galactose, which is present on the Fab portion of the Cetuximab heavy chain [38]. In order to overcome these severe hypersensitivity reactions which are observed in many immunoglobulin-based biotherapeutic agents it is of primary importance to ensure the oligosaccharide content will not elicit such reactions. Recently, a glyco-engineered anti-EGFR monoclonal antibody with a lower α-Gal content than Cetuximab was developed [41], highlighting the importance of these structures to the biopharma industry in the development of novel biotherapeutics.

In conclusion, while IgY is heavily decorated with complex glycans, no non-human immunogenic structures were identified. These results were determined using highly robust methods and are in accordance with previous IgY glycosylation studies from chicken serum [8]. The results from this study, combined with other known advantages of chicken antibodies, such as increased stability over IgG and phylogenetic distance from man [1], makes chickens ideal hosts for the generation of novel oral therapeutic interventions for the treatment of numerous infectious diseases.

## Supporting Information

S1 Fig Full IgY Sequence: (A) The complete amino acid sequence of IgY upsilon heavy chain including leader sequence and rearranged VDJ sequences and (B) The amino acid sequence chicken λ light chain. Numbering for chicken IgY heavy chain is based on the deduced amino acid sequences from cDNA, starting from the first alanine in the VH region [42,43] Numbering for chicken light chain immunoglobulin is derived from the nucleotide sequence from recombinant cDNA plasmids constructed from chicken spleen poly(A)-containing RNA [43]. (TIF)

S1 File Sequence alignment and methods for generation of homology model. (DOCX)

S1 Table Complete IgY *N-*glycan assignments. (DOCX)

S2 Table Summary of complete IgY *N-*glycan assignment and peak areas following excoglycosidase digestions. (XLSX)

## Abbreviations

2AB: 2-aminobenzamide
ABS: *Arthrobacter ureafaciens* sialidase
BEH: ethylenebridged hybrid
BKF: bovine kidney *α*-fucosidase
BTG: bovine testes *β*-galactosidase
EGFR: epidermal growth factor receptor
FLR: fluorescence
GU: glucose unit
GUH: *β-N-*Acetylglucosaminidase cloned from *Streptococcus pneumoniae*, expressed in *Escherichia coli*
HILIC: hydrophilic interaction liquid chromatography
HPLC: high-performance liquid chromatography
HRP: horse radish peroxidase
JBM: jack bean *α*-mannosidase
MS: mass spectrometry
MWCO: molecular weight cut-off
NAN1: *Streptococcus pneumoniae* sialidase
PA: *Pseudomonas aeruginosa*
PDB: protein data bank
PK: pharmacokinetic
PMT: photomultiplier
QTOF: quadrupole time of flight
SDS-PAGE: sodium dodecyl sulphate polyacrylamide gel electrophoresis
SPG: *Streptococcus pneumoniae β*-galactosidase
TMB: 3, 3', 5, 5'- tetramethylbenzidine
UPLC: ultra-performance liquid chromatography
WAX: weak anion exchange chromatography
