# Supplementary figures and images for: Comprehensive N-Glycan Profiling of Avian Immunoglobulin Y
Source: PLoS One. 2016 Jul 26;11(7):e0159859. doi: 10.1371/journal.pone.0159859 (PMC4961449; doi:10.1371/journal.pone.0159859)

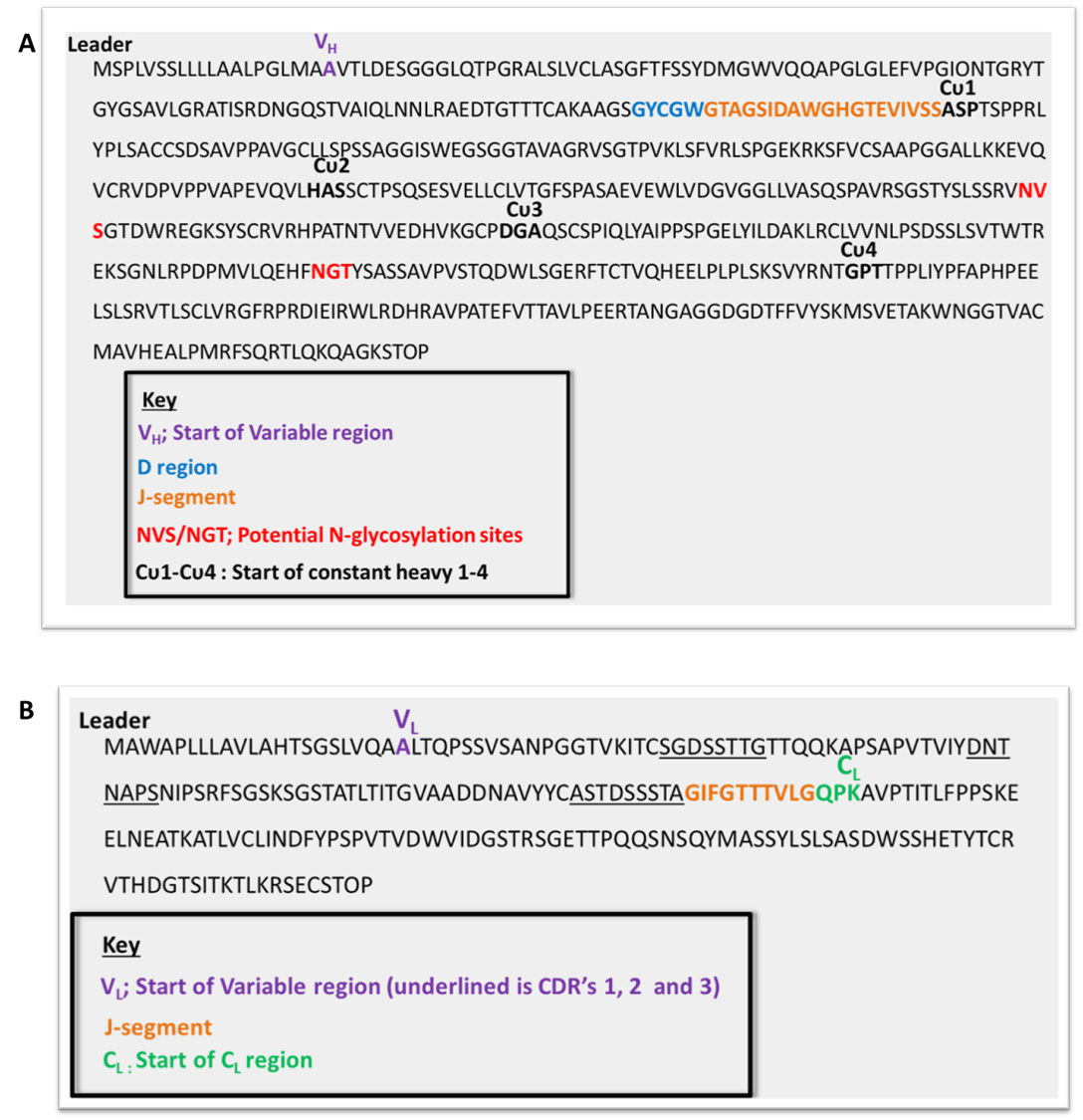

Supplement: S1 Fig — (TIF) [file pone.0159859.s001.tif]
