# Supplementary material for: Comprehensive N-Glycan Profiling of Avian Immunoglobulin Y
Source: PLoS One. 2016 Jul 26;11(7):e0159859. doi: 10.1371/journal.pone.0159859 (PMC4961449; doi:10.1371/journal.pone.0159859)
Supplement: S1 File — (DOCX) [file pone.0159859.s002.docx]

**Supplementary Information: Generation of IgY model:**

| Avian IgY  hIgE | AVTLDESGGGLQTPGRALSLVCLASGFTFSSYDMGWVQQAPGLGLEFVPGIONTGR  YTGYGSAVLGRATISRDNGQSTVAIQLNNLRAEDTGTTTCAKAAGSGYCGWGTAGS  IDAWGHGTEVIVSSASPTSPPRLYPLSACCSDSAVPPAVGCLLSPSSAGGISWEGS  GGTAVAGRVSGTPVKLSFVRLSPGEKRKSFVCSAAPGGALLKKEVQVCRVDPVPPV  DF  APEVQVLHASSCT--PSQSESVELLCLVTGFSPASAEVEWLVDGVGGLLVASQSPA  TPPTVKILQSSCDGGGHFPPTIQLLCLVSGYTPGTINITWLEDGQVMDVDLSTAST  VRSGSTYSLSSRVNVSGTDWREGKSYSCRVRHPATNTVVEDHVKGCPDGAQSCS*PI*  TQEGELASTQSELTLSQKHWLSDRTYTCQVTYQGH--TFEDSTKKCADS--NPRGV  *QLYAIPPSPGELYISLDAKLRCLVVNL-PSDSSLSVTWTREKSGNLRPDPMVLQEH*  SAYLSRPSPFDLFIRKSPTITCLVVDLAPSKGTVNLTWSRASGKPVNHSTRKEEKQ  *FNGTYSASSAVPVSTQDWLSGERFTCTVQHEELPLPLSKSVYRNTGPTTPPLIYPF*  RNGTLTVTSTLPVGTRDWIEGETYQCRVTHPHLPRALMRSTTKTSGPRAAPEVYAF  *APHPEELSLSRVTLSCLVRGFRPRDIEIRWLRDHRAVPATEFVTTAVLPEERTANG*  ATPEWPGSRDKRTLACLIQNFMPEDISVQWLHNEVQLPDARHSTTQPRKTK----G  *DGDTFFVYSKMSVETAKW-NGGTVACMAVHEAL-PMRFSQRTLQKQA*GK  SG--FFVFSRLEVTRAEWEQKDEFICRAVHEAASPSQTVQRAVSVNPG |
| --- | --- |

Avian IgY Sequence (Supplementary Figure 1): The section in italics corresponds to the sequence from the crystal structure 2w59.pdb- The crystal structure of avian IgY-Fc-3-4 Fragment.

hIgE – Sequence from the crystal structure 1o0v.pdb - The crystal structure of human IgE Fc.

The sequence alignment for the region PVAPEVQV … PDGAQSCS of IgY to hIgE was based on the Clustal alignment of the two sequences.

The sequence alignment for the region PIQLYAIP … RTLQKQAG of IgY to hIgE was performed by alignment of the three-dimensional structures (2w59 and 1o0v) in pymol.

The homology model was generated using: (i) a hIgG Fab domain crystal structure (for the IgY light chain and the IgY heavy chain sequence AVTLDESG … LKKEVQVC) which was linked to; (ii) a random coil sequence RVDPVP (for the IgY heavy chain linker region between domains Cu1 and Cu2) which was linked to; (iii) the hIgE Fc crystal structure (for the IgY heavy chain sequence PVAPEVQV … RTLQKQAG).
