# Supplementary material for: Comprehensive N-Glycan Profiling of Avian Immunoglobulin Y
Source: PLoS One. 2016 Jul 26;11(7):e0159859. doi: 10.1371/journal.pone.0159859 (PMC4961449; doi:10.1371/journal.pone.0159859)
Supplement: S1 Table — (DOCX) [file pone.0159859.s003.docx]

**Table 2: Summary of *N-*glycans identified from IgY purified form avian serum**

| **Peak** | **Structure** | | **Peak area (%)** | **GU** | **Experimental mass (m/z)** | **Theoretical mass (m/z)** | **Ion**  **Specie** |
| --- | --- | --- | --- | --- | --- | --- | --- |
| 5 | M4 | 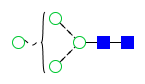 | 0.02 | 5.34 | --- | 1191.4421 | [M-H]^-^ |
| 9 | M5 | 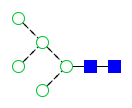 | 2.17 | 6.14 | 1353.514  676.2448 | 1353.4949  676.2438 | [M-H]^-^  [M-2H]^-2^ |
|  | FA2B | 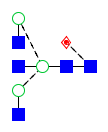 |  |  | 891.8257 | 891.8390 | [M-2H]^-2^ |
|  | FA3 | 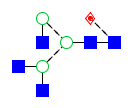 |  |  |  |  |  |
|  | A2[6]G1 | 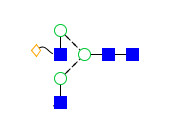 |  |  | 798.2899 | 798.2968 | [M-2H]^-2^ |
| 10 | A2[3]G1 | 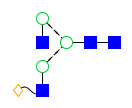 | 0.22 | 6.51 | 798.2899 | 798.2968 | [M-2H]^-2^ |
|  | A3G1 | 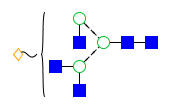 |  |  | 899.8270 | 899.8365 | [M-2H]^-2^ |
|  | A2BG1 | 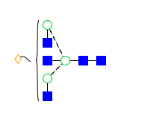 |  |  |  |  |  |
| 11 | M5A1 | 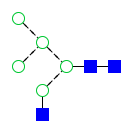 | 0.17 | 6.61 | 777.7769 | 777.7835 | [M-2H]^-2^ |
|  | M4A1G1 | 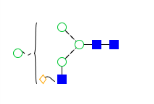 |  |  |  |  |  |

| **Peak** | **Structure** | | **Peak area (%)** | **GU** | **Experimental mass (m/z)** | **Theoretical mass (m/z)** | **Ion**  **Specie** |
| --- | --- | --- | --- | --- | --- | --- | --- |
| 12 | FA3G1 | 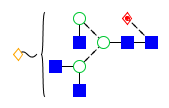 | 2.26 | 6.90 | 972.8573 | 972.8654 | [M-2H]^-2^ |
|  | FA2BG1 | 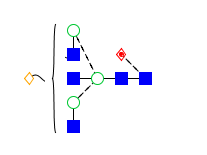 |  |  |  |  |  |
| 13 | FA3G1 | 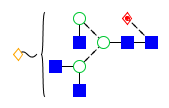 | 1.22 | 7.04 | 972.8509 | 972.8654 | [M-2H]^-2^ |
|  | FA2BG1 | 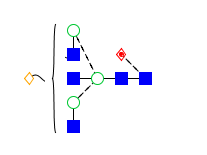 |  |  |  |  |  |
| 14 | M6 | 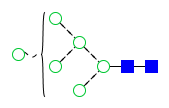 | 0.44 | 7.14 | 757.2596 | 757.2702 | [M-2H]^-2^ |
|  | A2G2 | 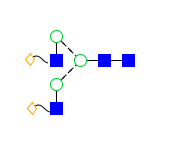 |  |  | 879.3184 | 879.3232 | [M-2H]^-2^ |
| 15 | A2BG2 | 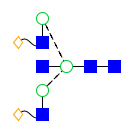 | 0.92 | 7.32 | 980.8661 | 980.8629 | [M-2H]^-2^ |
|  | A2[6]G1S1* | 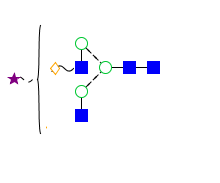 |  |  | 943.8293 | 943.8445 | [M-2H]^-2^ |

| **Peak** | **Structure** | | **Peak area (%)** | **GU** | **Experimental mass (m/z)** | **Theoretical mass (m/z)** | **Ion**  **Specie** |
| --- | --- | --- | --- | --- | --- | --- | --- |
| 16 | FA2G2 | 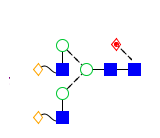 | 0.59 | 7.52 | 952.3262 | 952.3521 | [M-2H]^-2^ |
|  | M5A1G1 | 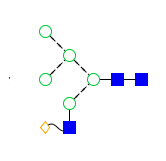 |  |  | 858.7934 | 858.8099 | [M-2H]^-2^ |
|  | A2[3]G1S1* | 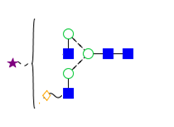 |  |  | 943.8293 | 943.8445 | [M-2H]^-2^ |
| 17 | FA2BG2 | 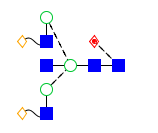 | 5.14 | 7.69 | 1053.8828 | 1053.8918 | [M-2H]^-2^ |
|  | FA3G2 | 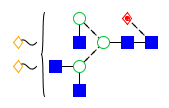 |  |  |  |  |  |
|  | A3G1S1* | 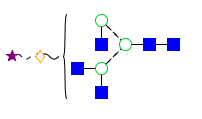 |  |  | --- | 1045.3842 | [M-2H]^-2^ |
|  | A2BG1S1* | 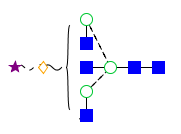 |  |  | --- | 1045.3842 | [M-2H]^-2^ |
|  | M4A1G1S1* | 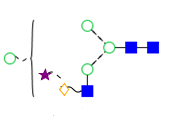 |  |  | 923.3214 | 923.3312 | [M-2H]^-2^ |

| **Peak** | **Structure** | | **Peak area (%)** | **GU** | **Experimental mass (m/z)** | **Theoretical mass (m/z)** | **Ion**  **Specie** |
| --- | --- | --- | --- | --- | --- | --- | --- |
| 18 | FA3’G2 | 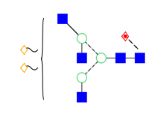 | 1.60 | 7.93 | 1053.8828 | 1053.8918 | [M-2H]^-2^ |
|  | FA2BG1S1* | 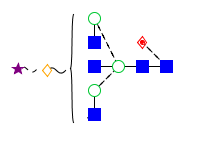 |  |  | 1118.4037 | 1118.4131 | [M-2H]^-2^ |
|  | FA3G1S1* | 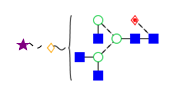 |  |  |  |  |  |
| 19 | M7 | 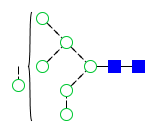 | 0.51 | 8.02 | 838.2908 | 838.2966 | [M-2H]^-2^ |
|  | FA2BG1S1* | 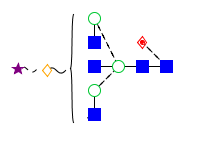 |  |  | 1118.4243 | 1118.4131 | [M-2H]^-2^ |
|  | FA3G1S1* | 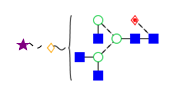 |  |  |  |  |  |
| 20 | A3G3 | 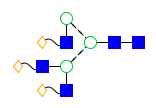 | 16.38 | 8.36 | --- | 1061.8893 | [M-2H]^-2^ |
|  | A2G2S1* | 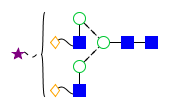 |  |  | 1024.8506 | 1024.8709 | [M-2H]^-2^ |
|  | A2G2S3* | 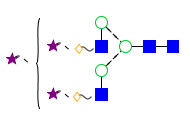 |  |  | --- | 1315.9663 | [M-2H]^-2^ |

| **Peak** | **Structure** | | **Peak area (%)** | **GU** | **Experimental mass (m/z)** | **Theoretical mass (m/z)** | **Ion**  **Specie** |
| --- | --- | --- | --- | --- | --- | --- | --- |
| 21 | A3BG3 | 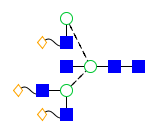 | 0.65 | 8.51 | --- | 1163.4290 | [M-2H]^-2^ |
|  | A2G2S3* | 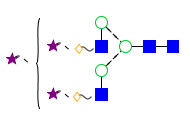 |  |  | --- | 1315.9663 | [M-2H]^-2^ |
|  | A2BG2S3* | 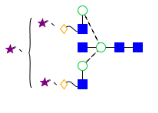 |  |  | --- | 1417.5060 | [M-2H]^-2^ |
| 22 | FA3G3 | 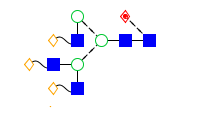 | 2.12 | 8.64 | 1134.9095 | 1134.9182 | [M-2H]^-2^ |
|  | A2BG2S1* | 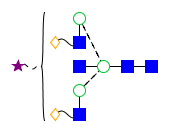 |  |  | 1126.4175 | 1126.4106 | [M-2H]^-2^ |
|  | A3’G3 | 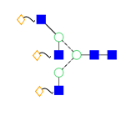 |  |  | --- | 1061.8893 | [M-2H]^-2^ |
| 23 | M8 | 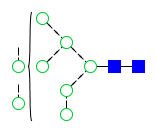 | 4.00 | 8.78 | 919.3203 | 919.3230 | [M-2H]^-2^ |
|  | FA2G2S1* | 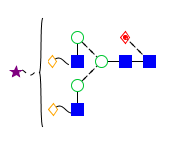 |  |  | 1097.8785 | 1097.8998 | [M-2H]^-2^ |
|  | M5A1G1S1* | 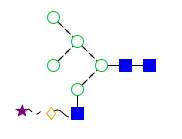 |  |  | 1004.3476 | 1004.3576 | [M-2H]^-2^ |
|  | FA2G2S3* | 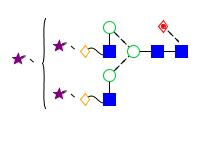 |  |  | --- | 1388.9953 | [M-2H]^-2^ |

| **Peak** | **Structure** | | **Peak area (%)** | **GU** | **Experimental mass (m/z)** | **Theoretical mass (m/z)** | **Ion**  **Specie** |
| --- | --- | --- | --- | --- | --- | --- | --- |
| 24 | FA3’G3 | 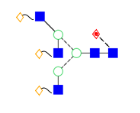 | 8.68 | 9.00 | 1134.9095 | 1134.9182 | [M-2H]^-2^ |
|  | FA2BG2S1* | 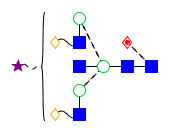 |  |  | 1199.4524 | 1199.4395 | [M-2H]^-2^ |
|  | FA3G2S1* | 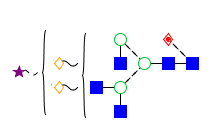 |  |  |  |  |  |
| 25 | FA4G3 | 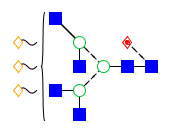 | 3.93 | 9.23 | --- | 1236.4579 | [M-2H]^-2^ |
|  | FA3’G2S1* | 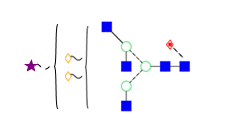 |  |  | 1199.4240 | 1199.4395 | [M-2H]^-2^ |
|  | A2G2S2* | 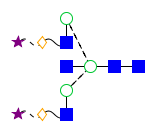 |  |  | 1170.4113 | 1170.4186 | [M-2H]^-2^ |
|  | FA2BG2S3* | 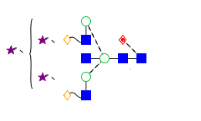 |  |  | --- | 1490.5349  993.3542 | [M-2H]^-2^  [M-3H]^-3^ |
|  | M5A1G1S3* | 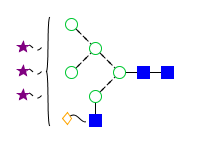 |  |  | --- | 1295.4530  863.2996 | [M-2H]^-2^  [M-3H]^-3^ |
|  | FA3G2S3* | 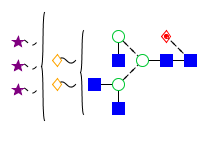 |  |  | --- | 1490.5349  993.3542 | [M-2H]^-2^  [M-3H]^-3^ |

| **Peak** | **Structure** | | **Peak area (%)** | **GU** | **Experimental mass (m/z)** | **Theoretical mass (m/z)** | **Ion**  **Specie** |
| --- | --- | --- | --- | --- | --- | --- | --- |
| 26 | M9 | 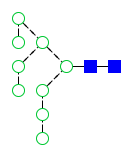 | 5.11 | 9.51 | 1000.3368 | 1000.3495 | [M-2H]^-2^ |
|  | A3G3S1* | 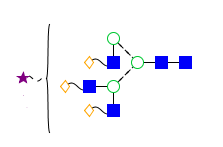 |  |  | 1207.4227 | 1207.4370 | [M-2H]^-2^ |
| 27 | A3BG3S1* | 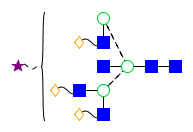 | 24.92 | 9.65 | --- | 1308.9767 | [M-2H]^-2^ |
|  | FA3’G2S3* | 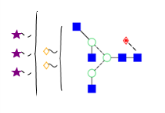 |  |  | --- | 1490.5349  993.3542 | [M-2H]^-2^  [M-3H]^-3^ |
|  | FA2BG1S4* | 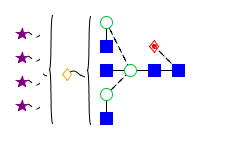 |  |  | --- | 1555.0562  1036.3684 | [M-2H]^-2^  [M-3H]^-3^ |
|  | FA3G1S4* | 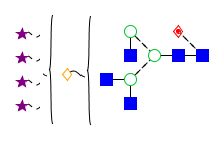 |  |  | --- | 1555.0562  1036.3684 | [M-2H]^-2^  [M-3H]^-3^ |
|  | A2G2S2* | 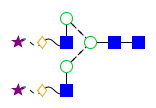 |  |  | 1170.4043 | 1170.4186 | [M-2H]^-2^ |

| **Peak** | **Structure** | | **Peak area (%)** | **GU** | **Experimental mass (m/z)** | **Theoretical mass (m/z)** | **Ion**  **Specie** |
| --- | --- | --- | --- | --- | --- | --- | --- |
| 28 | FA3G3S1* | 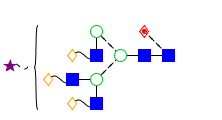 | 0.67 | 9.82 | --- | 1280.4659 | [M-2H]^-2^ |
|  | A4G4 | 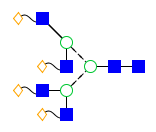 |  |  | 1244.4453 | 1244.4554 | [M-2H]^-2^ |
|  | A3’G3S1* | 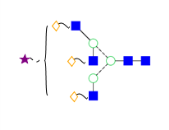 |  |  | 1207.4299 | 1207.4370 | [M-2H]^-2^ |
|  | A2BG2S2* | 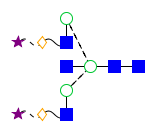 |  |  | --- | 1271.9583 | [M-2H]^-2^ |
|  | A4G4S4* | 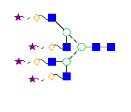 |  |  | --- | 1826.6462  1217.4284 | [M-2H]^-2^  [M-3H]^-3^ |
| 29 | FA3’G3S1* | 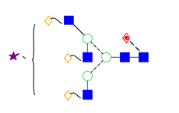 | 0.24 | 9.90 | --- | 1280.4659 | [M-2H]^-2^ |
|  | FA2G2S2* | 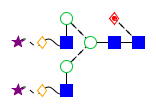 |  |  | 1243.4315 | 1243.4476 | [M-2H]^-2^ |
| 30 | FA4G4 | 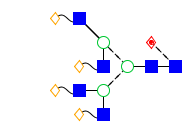 | 1.19 | 10.06 | --- | 1317.4843 | [M-2H]^-2^ |
|  | FA2BG2S2* | 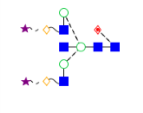 |  |  | 1344.9840 | 1344.9872 | [M-2H]^-2^ |

| **Peak** | **Structure** | | **Peak area (%)** | **GU** | **Experimental mass (m/z)** | **Theoretical mass (m/z)** | **Ion**  **Specie** |
| --- | --- | --- | --- | --- | --- | --- | --- |
| 31 | M9Glc | 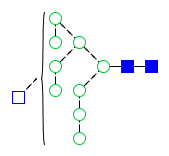 | 10.11 | 10.24 | 1081.3684 | 1081.3759 | [M-2H]^-2^ |
|  | FA3’G2S2* | 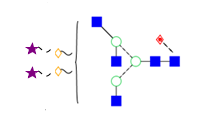 |  |  | 1344.9840 | 1344.9872 | [M-2H]^-2^ |
|  | FA2G2S4* | 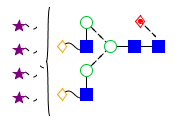 |  |  | --- | 1534.5430  1022.6929 | [M-2H]^-2^  [M-3H]^-3^ |
|  | A3G3S2* | 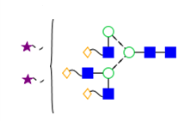 |  |  | 1352.9702 | 1352.9847 | [M-2H]^-2^ |
| 32 | A3BG3S2* | 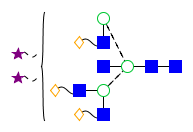 | 0.97 | 10.67 | --- | 1454.5244 | [M-2H]^-2^ |
| 33 | A3’G3S2* | 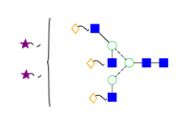 | 1.11 | 10.80 | 1352.9626 | 1352.9847 | [M-2H]^-2^ |
| 34 | FA3G3S2* | 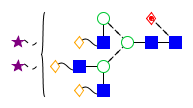 | 0.65 | 11.17 | --- | 1426.0137 | [M-2H]^-2^ |
|  | A3G3S3* | 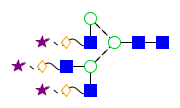 |  |  | 1498.5284  998.6880 | 1498.5324  998.6858 | [M-2H]^-2^  [M-3H]^-3^ |

| **Peak** | **Structure** | **Peak area (%)** | **GU** | **Experimental mass (m/z)** | **Theoretical mass (m/z)** | **Ion**  **Specie** |
| --- | --- | --- | --- | --- | --- | --- |

| 35 | FA3’G3S2* | 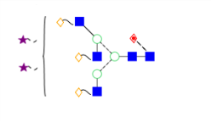 | 0.06 | 11.33 | --- | 1426.0137 | [M-2H]^-2^ |
| --- | --- | --- | --- | --- | --- | --- | --- |
|  | FA4G3S2* | 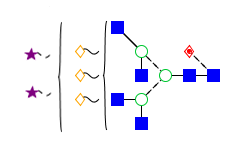 |  |  | --- | 1527.5533 | [M-2H]^-2^ |
|  | A3G3S3* | 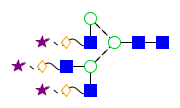 |  |  | 1498.5126  998.6880 | 1498.5324  998.6858 | [M-2H]^-2^  [M-3H]^-3^ |
|  | A3BG3S3* | 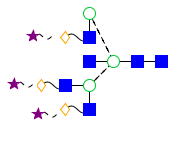 |  |  | --- | 1673.1010  1115.0649 | [M-2H]^-2^  [M-3H]^-3^ |
| 36 | A3G3S3* | 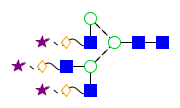 | 0.10 | 11.46 | 1498.5284  998.6880 | 1498.5324  998.6858 | [M-2H]^-2^  [M-3H]^-3^ |
|  | A4G4S2* | 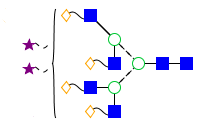 |  |  | 1535.5466 | 1535.5508 | [M-2H]^-2^ |
|  | A2G2S3* | 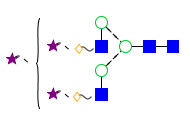 |  |  | --- | 1315.9663  876.9757 | [M-2H]^-2^  [M-3H]^-3^ |
| 37 | A2G2S3* | 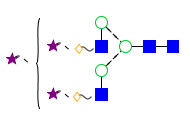 | 2.81 | 11.59 | --- | 1315.9663  876.9757 | [M-2H]^-2^  [M-3H]^-3^ |
|  | A3G3S3* | 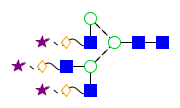 |  |  | 1498.5284  998.6880 | 1498.5324  998.6858 | [M-2H]^-2^  [M-3H]^-3^ |

| **Peak** | **Structure** | **Peak area (%)** | **GU** | **Experimental mass (m/z)** | **Theoretical mass (m/z)** | **Ion**  **Specie** |
| --- | --- | --- | --- | --- | --- | --- |

| 38 | A3’G3S3* | 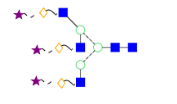 | 0.13 | 11.92 | --- | 1498.5324  998.6858 | [M-2H]^-2^  [M-3H]^-3^ |
| --- | --- | --- | --- | --- | --- | --- | --- |
|  | A3BG3S3* | 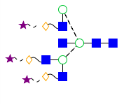 |  |  | --- | 1600.0721  1066.3790 | [M-2H]^-2^  [M-3H]^-3^ |
|  | A2BG2S3* | 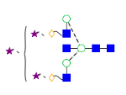 |  |  | --- | 1417.5060  944.6682 | [M-2H]^-2^  [M-3H]^-3^ |
|  | FA3G3S3* | 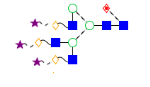 |  |  | --- | 1571.5614  1047.3718 | [M-2H]^-2^  [M-3H]^-3^ |
| 39 | FA2G2S3* | 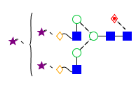 | 0.52 | 12.09 | --- | 1388.9953  925.6611 | [M-2H]^-2^  [M-3H]^-3^ |
|  | FA3G3S3* | 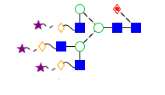 |  |  | --- | 1571.5614  1047.3718 | [M-2H]^-2^  [M-3H]^-3^ |
| 40 | M5A1G1S3* | 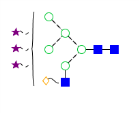 | 0.10 | 12.66 | --- | 1295.4530  863.2996 | [M-2H]^-2^  [M-3H]^-3^ |
|  | A3'G3S3* | 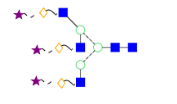 |  |  | 1498.5284  998.6880 | 1498.5324  998.6858 | [M-2H]^-2^  [M-3H]^-3^ |
|  | A4G4S3* | 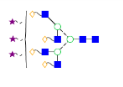 |  |  | --- | 1681.0985  1120.3966 | [M-2H]^-2^  [M-3H]^-3^ |
|  | FA2BG2S3* | 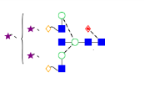 |  |  | --- | 1490.5349  993.3542 | [M-2H]^-2^  [M-3H]^-3^ |
|  | A3G3S4* | 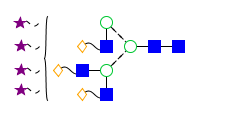 |  |  | --- | 1644.0801  1095.7177 | [M-2H]^-2^  [M-3H]^-3^ |

Table of *N*-glycans released from IgY purified from serum. The HILIC-chromatogram was separated into 40 peaks and structural assignment carried made using established methods (Royle et al., 2008) and the software tool GlycoBase (https://glycobase.nibrt.ie). *Sialic acid linkages; WAX fractions were separated out into 5 fractions; S1: Monosialylated, S2: Disialylated, S3: Trisialylated, S3J: Trisialylated and S4: Tetrasialylated. All monosialylated glycans are linked by α2-3 and α2-6; Disialylated glycans have all combinations of α2-3 and α2-6 linkages [i.e. (3, 3), (3, 6) and (6, 6)]; Trisialylated glycans have all combinations of α2-3 and α2-6 linkages except (6, 6, 6) [i.e. (3, 3, 3), (3, 3, 6) and (3, 6, 6)]; and Tetrasialylated glycans have all combinations of α2-3 and α2-6 linkages [i.e. (3,3,3,3), (3,3,3,6) , (3,3,6,6) and (6,6,6,6)]. Structure abbreviations: all *N*-glycans have two core GlcNAcs; F at the start of the abbreviation indicates a core-fucose α1,6-linked to the inner GlcNAc; Mx, number (x) of mannose on core GlcNAcs; Ax, number of antenna (GlcNAc) on trimannosyl core; A2, biantennary with both GlcNAcs as β1,2-linked; A3, triantennary with a GlcNAc linked β1,2 to both mannose and the third GlcNAc linked β1,4 to the α1,3 linked mannose; A3’ , isomer with the third GlcNAc linked β1-6 to the α1-6 linked mannose; A4, GlcNAcs linked as A3 with additional GlcNAc β1,6 linked to α1,6 mannose; B, bisecting GlcNAc linked β1,4 to β1,3 mannose; Gx, number (x) of β1,4 linked galactose on antenna; F(x), number (x) of fucose linked α1,3 to antenna GlcNAc; Sx, number (x) of sialic acids linked mostly to galactose (Structures with more sialic acids then galactoses may have some sialic acids linked to GlcNAc or another sialic acid in form of polysialic acid.).
